# Identification of photoperiod-induced specific miRNAs in the adrenal glands of Sunite sheep (*Ovis aries*)

**DOI:** 10.3389/fvets.2022.888207

**Published:** 2022-07-22

**Authors:** Xiaolong Du, Xiaoyun He, Qingqing Liu, Qiuyue Liu, Ran Di, Mingxing Chu

**Affiliations:** ^1^Key Laboratory of Animal Genetics, Breeding and Reproduction of Ministry of Agriculture and Rural Affairs, Institute of Animal Sciences of Chinese Academy of Agricultural Sciences, Beijing, China; ^2^College of Animal Science and Technology, Anhui Agricultural University, Hefei, China

**Keywords:** sheep, adrenal gland, photoperiod, reproduction, RNA-seq

## Abstract

In seasonal estrus, it is well known that melatonin-regulated biorhythm plays a key role. Some studies indicate that the adrenal gland plays an important role in reproduction in mammals, but the molecular mechanism is not clear. This study used an artificially controlled light photoperiod model, combined with RNA-seq technology and bioinformatics analysis, to analyze the messenger RNA (mRNA) and microRNA (miRNA) of ewe (Sunite) adrenal glands under different photoperiod treatments. After identification, the key candidate genes *GRHL2, CENPF, FGF16* and *SLC25A30* that photoperiod affects reproduction were confirmed. The miRNAs (oar-miR-544-3p, oar-miR-411b-5p, oar-miR-376e-3p, oar-miR-376d, oar-miR-376b-3p, oar-miR-376a-3p) were specifically expressed in the adrenal gland. The candidate mRNA-miRNA pairs (e.g., *SLC25A30* coagulated by novel miRNA554, novel miRNA555 and novel miRNA559) may affect seasonal estrus. In summary, we constructed relation network of the mRNAs and miRNAs of sheep adrenal glands using RNA sequencing and bioinformatics analysis, thereby, providing a valuable genetic variation resource for sheep genome research, which will contribute to the study of complex traits in sheep.

## Introduction

Low fecundity is the greatest limiting factor in the modern mutton sheep industry. Many element affect fecundity, such as litter size, oestrus frequency, and embryo survival rate. Because the most part area of China is located in the temperate zone, many animals are in oestrus in autumn and winter and give birth in spring and summer to ensure the survival of their offspring. Biologists are therefore divided into long-photoperiod breeds (LPs) and short-photoperiod breeds (SPs) with seasonal variations (1, 2). For example, the Chinese Sunite sheep is a typical SP breeder, which is specifically represented as estrus from August to March of the next year and anestrus from April to July (3, 4).

Concerning reproduction, the unavoidable theme is the upstream control center, such as the hypothalamus and pituitary gland. Studies have shown that light stimulates the paraventricular nucleus (PVN) and then affects the production of melatonin in the pineal gland, ultimately acting on the hypothalamus and affecting the reproduction of sheep through the hypothalamus-pituitary-gonad axis (HPG) (5–9). In addition, stimulating PVN also secretes corticotropin-releasing hormone (CRH), which then activates the pituitary gland to release corticotropin (ACTH). ACTH in turn stimulates the adrenal gland to secrete cortisol, which then provides negative feedback to the brain in the classic steady-state feedback loop to regulate hypothalamus-pituitary-adrenal gland axis (HPA) signals (10). However, the close relationship between HPA and HPG is more than that. When the fetus develops in the uterus, the hormone system that regulates the HPG and HPA axes plays an important role in the growth and development of its tissue. Fetal glucocorticoid concentrations increase in the third trimester of pregnancy, which is conducive to the modification of fetal key tissues or organs to promote fetal survival, including lung maturation and pulmonary surfactant production (11). In a study of rodents, exposure to glucocorticoids was found to closely affect fetal development during intrauterine development, such as gonadogenesis, the establishment of the HPG axis and the reproductive tract's morphogenesis (12, 13). In addition, the increase in the concentration of glucocorticoids interferes with the concentration of serum testosterone (T) because it inhibits T biosynthesis (14).

With the development of high-throughput sequencing, RNA sequencing (RNA-seq) is increasingly widely used in livestock. Including sheep (15) and cattle (16), this technique is used to obtain the expression profile information of mRNA and miRNA, which makes a great contribution to revealing some important traits and mining their candidate genes (17). Research on miRNAs has shown a trend of the great outbreak in recent years. Precursor miRNAs are well known to be transcribed mainly by RNA polymerase II, digested by dicer and then processed into mature miRNAs (18). Many studies have shown that miRNA plays a key role in regulating animal phenotype, such as affecting wool curvature (19), immunity to infectious diseases (20) and fat deposition (21). In this study, we mainly explored the key miRNAs affecting reproductive traits and adrenal-specific expression of miRNAs in sheep adrenal tissue under different photoperiod conditions. Finally, the relationship between differentially expressed mRNAs and miRNAs was predicted by bioinformatics software, and an interaction network is constructed, which is expected to mine effective information.

## Materials and methods

### Ethics statement

The experimental animals involved in this study were carried out after being examined by the Animal Experimental Welfare Ethics Committee of the Institute of Animal Sciences of Chinese Academy of Agricultural Sciences (IAS-CAAS, Beijing, China). In addition, the review acceptance number is No. IAS2018-3, and all the experimental procedures are executed in accordance with the relevant guidelines and regulations formulated by the Ministry of Agriculture and Rural Affairs of the People's Republic of China.

### Preparation of animals

Nine non-pregnant adult Sunite ewes (aged 2–3 years old; weight 30–40 kg), which were randomly selected from a farm in Bayan Nur City (40°75′north latitude), Inner Mongolia Autonomous Region, China, were used for the study. The selected ewes were uniformly transported to a farm at the Tianjin Institute of Animal Sciences, Tianjin (39°13′ north latitude), China, and the follow-up experiments were carried out after a month of routine feeding to adapt to the local environment. The ovaries of these animals were removed by surgery, and an estrogen silicone tube (E_2_, Sigma Chemical Co., St. Louis, MO, USA) was implanted subcutaneously in the neck of the sheep to maintain plasma estradiol levels of 3–5 pg/ml, as described previously (1, 22, 23). The ewe postoperative recovery lasted for 30 days before artificial light period control. The ewes were randomly divided into three groups: SP42 (short photoperiod for 42 days; *n* = 3), LP42 (long photoperiod for 42 days; *n* = 3) and SPLP42 (short photoperiod for 42 days followed by a long photoperiod for 42 days; *n* = 3). The conditions for the long photoperiod were 16 h of light per day and 8 h without light. The lighting duration setting for the short photoperiod was the opposite of the lighting duration setting for the long photoperiod. All sheep had *ad libitum* feeding and drinking in an enclosed climate control chamber with only artificial light sources.

### Tissues acquisition, library construction and sequencing

Adrenal gland tissue from euthanized ewes was quickly preserved in liquid nitrogen. Then, the stored tissues were used for RNA extraction with TRIzol Reagent (Invitrogen, Carlsbad, CA, USA) according to the manufacturer's instructions.

The mRNA library was constructed with 3 μg of high-quality RNA using the NEB Next Ultra Directional RNA Library Prep Kit for Illumina (NEB, Ipswich, USA) according to the manufacturer's instructions. During this process, Ribo-Zero™ Gold Kits (TIANGEN, Beijing, China) were used to remove rRNA. In addition, we used the UNG enzyme to degrade the second strand of U-containing cDNA and performed polymerase chain reaction (PCR) amplification to obtain an RNA library. Then, a PE150 (paired-end 150 bp, PE150) sequencing approach for mRNAs was performed on a Hiseq X platform (Illumina, San Diego, CA, USA).

The miRNA library was built by a Small RNA Sample Pre kit (TIANGEN). We directly took total RNA as the starting sample, added adaptors at both ends of small RNA, and then reverse-transcribed the RNA to synthesize cDNA. After PCR amplification, the target DNA fragments were separated by polyacrylamide gel electrophoreses (PAGE), and the 140- to 160-bp ligation products were recovered to generate a cDNA library. In addition, an SE50 (single-end 50 bp, SE50) sequencing approach for miRNAs was performed on the Illumina HiSeq2500 platform (Illumina, San Diego, CA, USA). All sequencing data were outsourced to Annoroad Gene Technology Co., Ltd. (Beijing, China).

### Data processing and transcriptome assembly

Bcl2fastq (v2.17.1.14) was used to process the offline data and convert the original image file into row sequencing reads on-base calling, which were row reads. Using an in-house Perl script made by Annoroad Genentech Co., Ltd. (Beijing, China) to remove low-quality reads, reads with adaptor contamination and reads with a rate of N > 5%, the clean mRNA reads were acquired from the raw reads. We used the Ovis aries reference genome (Oar_v4.0) and the genome annotation file from Ensembl. Cleaned reads were then mapped to the reference genome using HiSAT2 (v2.0.5) (24), and StringTie (v1.3.2d) was used to assemble the transcripts (25). HiSAT2 was run with “-rna - strandness RF” and “-dta -t -p 4,” String Tie with “-G ref.gtf -rf−1,” and the other parameters were set as the default. In addition to the above steps, the following steps were added to obtain the clean miRNA reads. These steps include removing reads without a 3'adaptor and insert fragment, removing the reads containing consecutive A/T/G/C bases, and removing the reads with abnormal final length. To ensure the accuracy of the subsequent analysis, the clean reads of sRNA sequencing were mapped to the reference genome (Oar_v4.0) by the comparison analysis software Bowtie v1.1.2.

### Classification notes of sRNA and identification of miRNA

We obtained the situation, in which the sequence matched different regions in each sample by mapping the clean reads to the Ovis aries sequence in the miRBase database (Release 21) (26). At the same time, the known miRNA can be identified. The clean reads that were not annotated as a known miRNA were compared with the ncRNA sequence in Rfam (13.0) (27) to realize the annotation of rRNA, tRNA, snRNA, snoRNA and other ncRNA. The RepeatMasker program was used to comment on different types of repeats for clean reads that were not annotated as known miRNAs and ncRNAs (28). After identifying the above sRNA types and then using the matching (100% positional overlap) results with the location information of exons and introns of the gene, the sRNA from mRNA will be annotated (29). For sRNA reads that did not match the above-known annotation type, the software miRDeep2 (30) was used to predict the new miRNA, and the sequence, expression and structure information of each new miRNA were analyzed. Different mature body sequences, precursor sequences and positions will be considered different new miRNAs.

### Differential expression and functional enrichment analysis of miRNA and mRNAs

The transcripts per million (TPM) and the fragments per kilobase per million mapped reads (FPKM) values were calculated to represent miRNA and mRNA expression levels based on the read number (31). The difference analysis was carried out by the software DESeq (32). The screening conditions of differentially expressed miRNAs were pval ≤ 0.05, padj ≤ 0.05 and log_2_| (fold change) | ≥1. The differential expression criteria of mRNA were |log_2_Ratio| ≥1 and *q* < 0.05. In addition, the target genes of known and novel differentially expressed miRNAs were predicted by miRanda and TargetScan software, respectively (33). The intersection of the prediction results of the two software programs was selected as the target gene of the miRNAs.

We performed Gene Ontology (GO) and Kyoto Encyclopedia of Genes and Genomes (KEGG) analyses based on the targeted genes of differentially expressed miRNAs (DEMs) and differentially expressed mRNAs (DEGs). The hypergeometric test method was applied to assess significantly enriched GO terms and KEGG pathways, and FDR <0.05 was considered to be significantly enriched.

### Construction of integral miRNA–mRNA interaction networks

To further describe the interaction between miRNA and mRNA, we used miRanda and TargetScan software for prediction and took the intersection of the prediction results of the two software programs as the target genes of miRNA. According to the identified DEMs and differentially expressed target mRNAs of DEMs, a regulatory network diagram was drawn by Cytoscape software (34).

### Data validation

Transcripts (*n* = 4) were randomly selected and the primers were designed by Primer 5.0 software. The miRNA primers were synthesized by Shanghai Sangon Biotech. The miRNA quantitative (q)PCR conditions were as follows: 95°C for 15 min, followed by 40 cycles of 94°C for 20 s and 60°C for 34 s (miRcute Plus miRNA qPCR Kit, TIANGEN). In addition, U6 small nuclear RNA (snRNA; for miRNA) were utilized as reference genes. The data obtained from the qPCR were evaluated using the 2^−Δ*ΔCt*^ method.

## Results

### Overview of sequencing data in adrenal gland tissue

We obtained the global expression profile of mRNA and miRNA in the adrenal glands of Sunite ewes under different photoperiod treatments. The data of subsequent analysis, namely, clean reads, were based on the filtered original offline data, which had no adapter-polluted reads, and the base error rate was <0.1%. Overall, we obtained 1,029 million clean reads of mRNA and 244 million clean reads of miRNA. To obtain more accurate sequences and the accuracy of subsequent analysis, bioinformatics analysis software was used to map clean reads to the reference genome and compare the results statistically. The results are shown in Table 1. We found that the average comparison rate of miRNA was ~50%, while the average comparison rate of mRNA was approximately 95%. In addition, 123 million perfect match reads of miRNA and 978 million perfect match reads of mRNA were obtained separately (Table 1; Supplementary Table 1).

**Table 1:** Summary of the mapping data from the adrenal gland tissues.

**Sample**	**Total reads**	**Mapped reads**	**Mapping rate (%)**	**Total reads**	**Mapped reads**	**Mapping rate (%)**
	**miRNA**			**mRNA**		
LP42-1	28587504	13967493	48.86	111648952	105998978	94.94
LP42-2	27102088	12510029	46.16	101268666	95551270	94.35
LP42-3	27778108	14175856	51.03	111612316	106337978	95.27
SP42-1	20353895	10420944	51.2	125490436	119791918	95.46
SP42-2	30012282	15368864	51.21	110469132	104921437	94.98
SP42-3	26406199	13258597	50.21	115155856	109685478	95.25
SPLP42-1	24951610	12495852	50.08	121476492	115054106	94.71
SPLP42-2	29122928	14768748	50.71	120023448	114183042	95.13
SPLP42-3	29856881	16144823	54.07	111722560	106093325	94.96
Total	244171495	123111206		1028867858	977617532	

*LP42, and SP42 represent a long light period every day for 42 days and a short light period every day for 42 days. SP-LP42 means a short light period every day for 42 days, followed by a long light period for 42 days*.

### Identification of mRNAs and miRNAs in adrenal gland tissue

**Figure 1:**
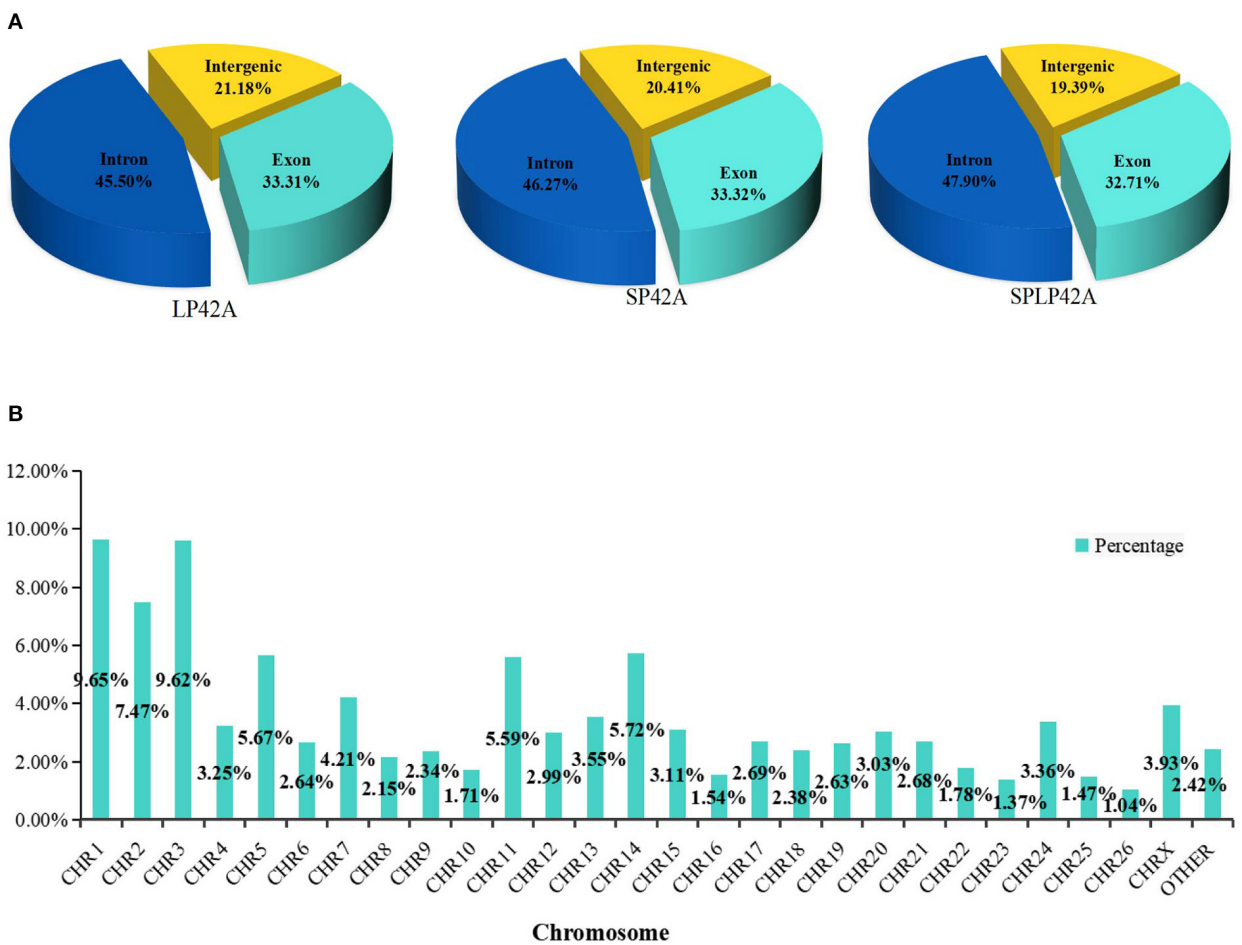
Summary information of mRNA identification. **(A)** According to the sequence alignment information, the number of sequences aligned to exons, introns and intergenic regions was calculated, and the pie chart was made according to the proportion. **(B)** Chromosome distribution of identified genes from the adrenal gland.

A total of 18,947 mRNAs were identified (Supplementary Table 2) after mapping to the sheep genome. According to the *Ovis aries* gene annotation files in the related database, we counted the number and proportion of the unique alignment sequences on the three functional elements of the gene (exon, intron and intergenic). In general, if the annotation information of the species is more comprehensive, most of the sequences should be aligned to the exon region, but alternative splicing and noise expression will cause some sequences to come from the intron region and the intergenic region. The following figure shows the distribution of unique alignment sequences in various regions of the genome and chromosome. Our results suggested that many mRNAs were situated in the intergenic region (more than 45%), followed by the exon (approximately 33%) and intron (nearly 20%) regions (Figure 1A; Supplementary Table 3). Furthermore, the chromosome distribution of mRNAs showed that chromosome 3 and chromosome 1 expressed the most genes, accounting for 9.62 and 9.65%, respectively, followed by chromosome 2 (7.47%) and chromosome 5 (5.67%) (Figure 1B; Supplementary Table 4).

**Figure 2:**
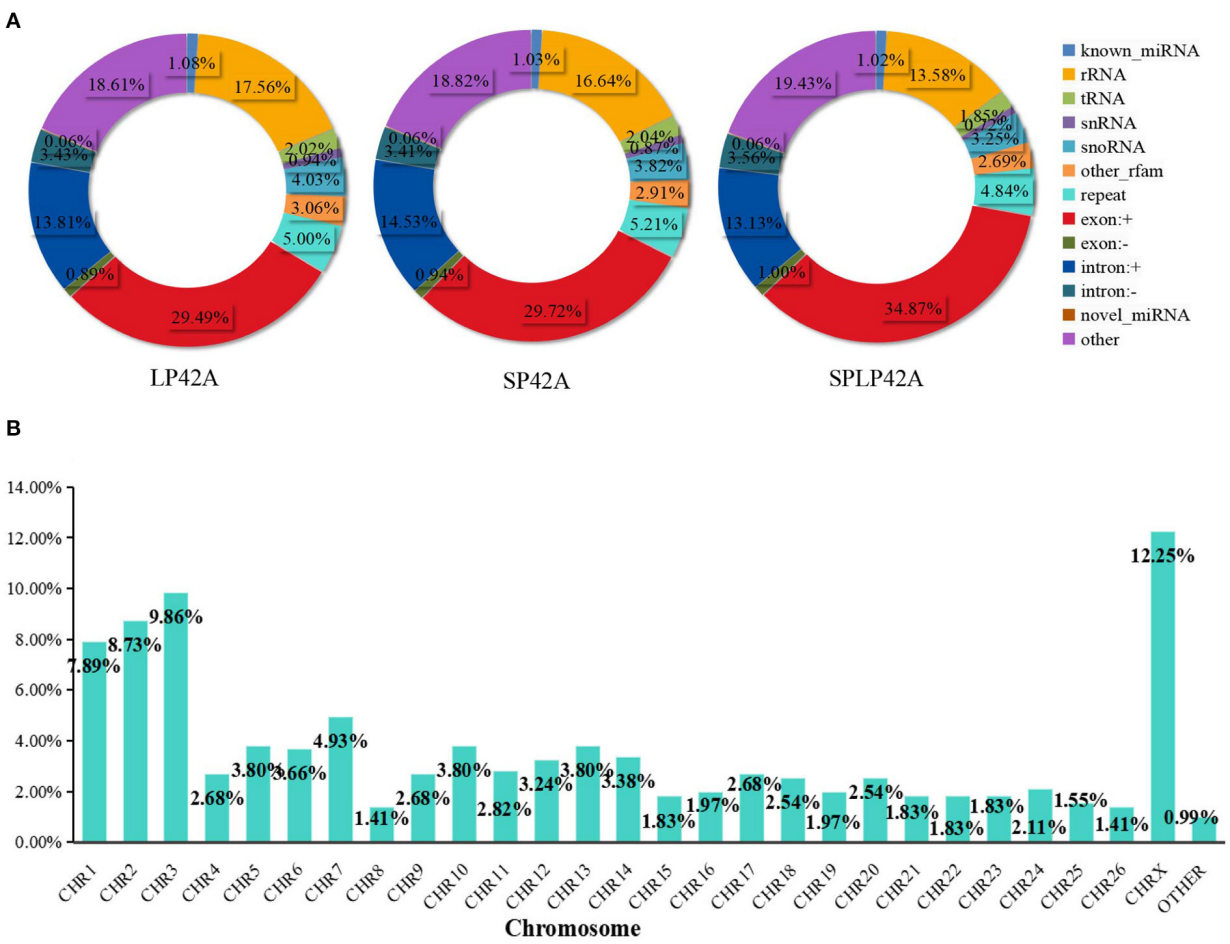
Summary information of sRNA identification. **(A)** The statistical results of unique clean reads from the adrenal gland in the three experimental groups. **(B)** Chromosome distribution of identified miRNA from the adrenal gland.

Regarding miRNAs, RNA-seq generated approximately 244 million clean reads after filtering and mapping. We classified and annotated all clean reads, including known miRNAs, ncRNAs (rRNA\ tRNA\ snRNA\ snoRNA\ other rfam), repeats, and small RNAs annotated by perfect matching with mRNA exons and introns and novel miRNAs. The detailed classification and specific statistics are shown in Table 2. The proportion of clean reads matching known miRNA maturity in each sample was more than 70%, followed by the proportion of clean reads matching with other ncRNA in the rfam database being more than 15%. In addition, we also counted the number of clean read types that matched the miRNA maturity, namely, unique clean reads. The statistical results are shown in Figure 2A. We found that the following four types, namely, exon+, other, rRNA and intron+, accounted for more than 10% of unique clean reads in the three experimental groups. Compared with the statistical results of total reads, the proportion of unique clean reads with known miRNA typeS decreased significantly, from ~70 to 1% (Supplementary Table 6). The distribution of chromosomes is similar to the distribution of chromosomes of mRNA. The results of Figure 2B show that the X chromosome contains the most miRNA (12.25%), followed by chromosomes 3 (9.86%), 2 (8.73%) and 1 (7.89%) (Supplementary Table 4).

**Table 2:** Summary of the classification statistics of sRNA total reads mapped to reference genome.

**Types**	**LP42**	**Proportion**	**SP42**	**Proportion**	**SPLP42**	**Proportion**
Total	40653378	100%	39048405	100%	43409423	100%
Known miRNA	29903694	75.20%	29418632	73.61%	34055160	78.20%
rRNA	952232	2.18%	879015	2.37%	664044	1.50%
tRNA	522878	2.67%	947718	1.29%	495703	1.18%
snRNA	16647	0.03%	12853	0.04%	11207	0.02%
snoRNA	820367	2.06%	786458	2.02%	690074	1.59%
Other rfam	7321624	15.05%	5898519	17.92%	6508957	15.22%
Repeat	93962	0.20%	79954	0.23%	83080	0.19%
Exon:+	210897	0.53%	207450	0.52%	244056	0.57%
Exon:−	10167	0.03%	10336	0.03%	10320	0.02%
Intron:+	287885	0.70%	275741	0.71%	261042	0.61%
Intron:−	46007	0.20%	86753	0.12%	39145	0.09%
Novel miRNA	52460	0.12%	46912	0.13%	77845	0.18%
Other	414558	1.04%	398064	1.02%	268790	0.63%

*The data of LP42, SP42 and SPLP42 are the total number of three biological repetitive samples, respectively. All the data and proportion calculations are based on mapped reads*.

**Figure 3:**
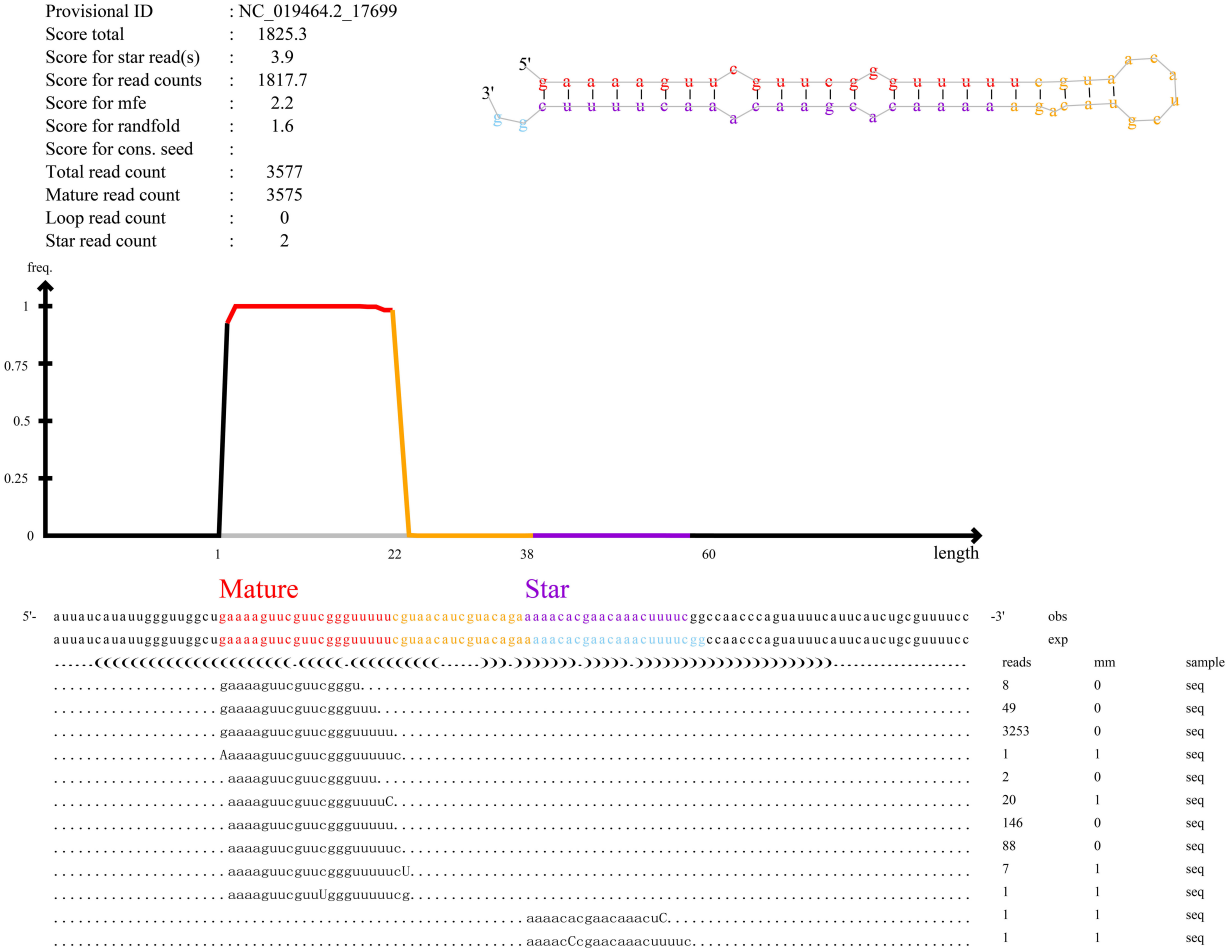
An example of the miRDeep2 output. The figure illustrates the output for the miRNA of novel_555. The upper part of Figure 3 shows the read count and the total count of each part. The predicted secondary structure of the hairpin is also depicted, with mature (red), star (purple) and circular (yellow) sequences highlighted in different colors. The bottom of the map shows the results of miRNA alignment with predicted precursor sequences on the genome (OBS line) and experimental sequences reported in miRBase (EXP line). For each sequence, the frequency and mismatch with the genome sequence (mm sequence) are given. Mismatches are also highlighted in uppercase letters.

Furthermore, our main concern is the identification of known miRNAs and novel miRNAs. In total, we identified miRNAs (*n* = 861), including known miRNAs (*n* = 151) and novel miRNAs (*n* = 710). The numbers of total clean reads identified as known miRNAs and novel miRNAs in the three experimental group samples were 93,377,486 and 177,217, respectively, and the numbers of unique clean reads identified as known miRNAs and novel miRNAs in the samples were 18,691 and 1,054, respectively. Figure 3 shows an example of miRDeep2 output. The top of Figure 3 shows the scores assigned to each part of the miRNA and the total count. Different colors are used to represent different parts of the predicted hairpin secondary structure. All readings related to miRNA are also shown at the bottom of the figure. Therefore, about the detailed miRDeep2 output information of novel miRNAs, we described in detail the three biological repetitive samples of the three processing groups (LP42-1 *n* = 115, LP42-2 *n* = 120, LP42-3 *n* = 119, SP42-1 *n* = 94, SP42-2 *n* = 138, SP42-3 *n* = 110, SPLP42-1 *n* = 117, SPLP42-2 *n* = 120, SPLP42-3 *n* = 121), including the precursor secondary structure, count number and read sequence shown in the picture. Known miRNA is also described in detail (LP42-1 *n* = 106, LP42-2 *n* = 106, LP42-3 *n* = 106, SP42-1 *n* = 104, SP42-2 *n* = 106, SP42-3 *n* = 105, SPLP42-1 *n* = 106, SPLP42-2 *n* = 106, SPLP42-3 *n* = 106). The known miRNA specifically expressed in the adrenal gland was found by comparing with Zhang's Small Tail Han sheep hypothalamic miRNA transcriptome data (17), only six miRNAs (oar-miR-544-3p, oar-miR-411b-5p, oar-miR-376e-3p, oar-miR-376d, oar-miR-376b-3p, oar-miR-376a-3p) were specifically expressed in the adrenal gland, and one (oar-miR-1193-3p) was specifically expressed in the hypothalamus (Supplementary Tables 5, 6, miRDeep2 file).

### The analysis of differentially expressed miRNAs and mRNAs in adrenal gland tissue

**Figure 4:**
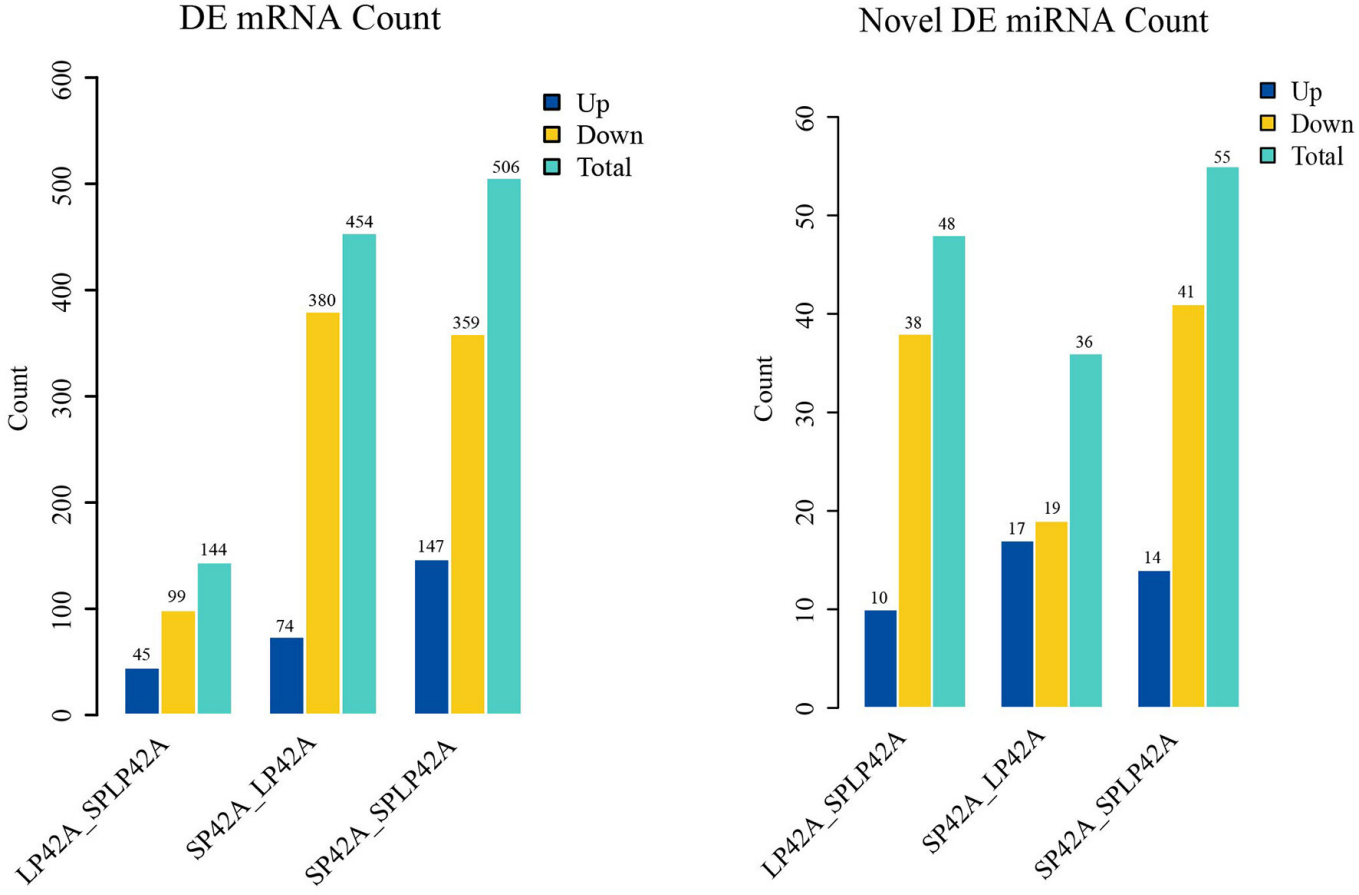
Differentially expressed (DEM) microRNA and (DEG) mRNA analysis. Different color rectangles were used to represent the numbers of upregulated (blue), downregulated (yellow) and total (green) differential genes.

The numbers of DEGs and DEMs identified from the LP42 vs. SPLP42 comparison group were 144 and 48, respectively. Among these DEGs and DEMs, 45 and 10 genes were upregulated, and 99 and 38 genes were downregulated. In the same way, we counted the DEGs and DEMs of the SP42 vs. LP42 (DEG n = 454, DEM n = 36) and SP42 vs. SPLP42 (DEG *n* = 506, DEM *n* = 55) comparison groups. After analyzing the differences between all known miRNAs and novel miRNAs, we found that in known miRNAs, only the oar-miR-148a of the LP42 vs. SPLP42 comparison group was differentially expressed, so the graph shows only the statistics of novel miRNAs. Overall details are shown in Figure 4; Supplementary Table 7.

### GO and KEGG enrichment analysis of DEGs and target gene of DEMs

**Figure 5:**
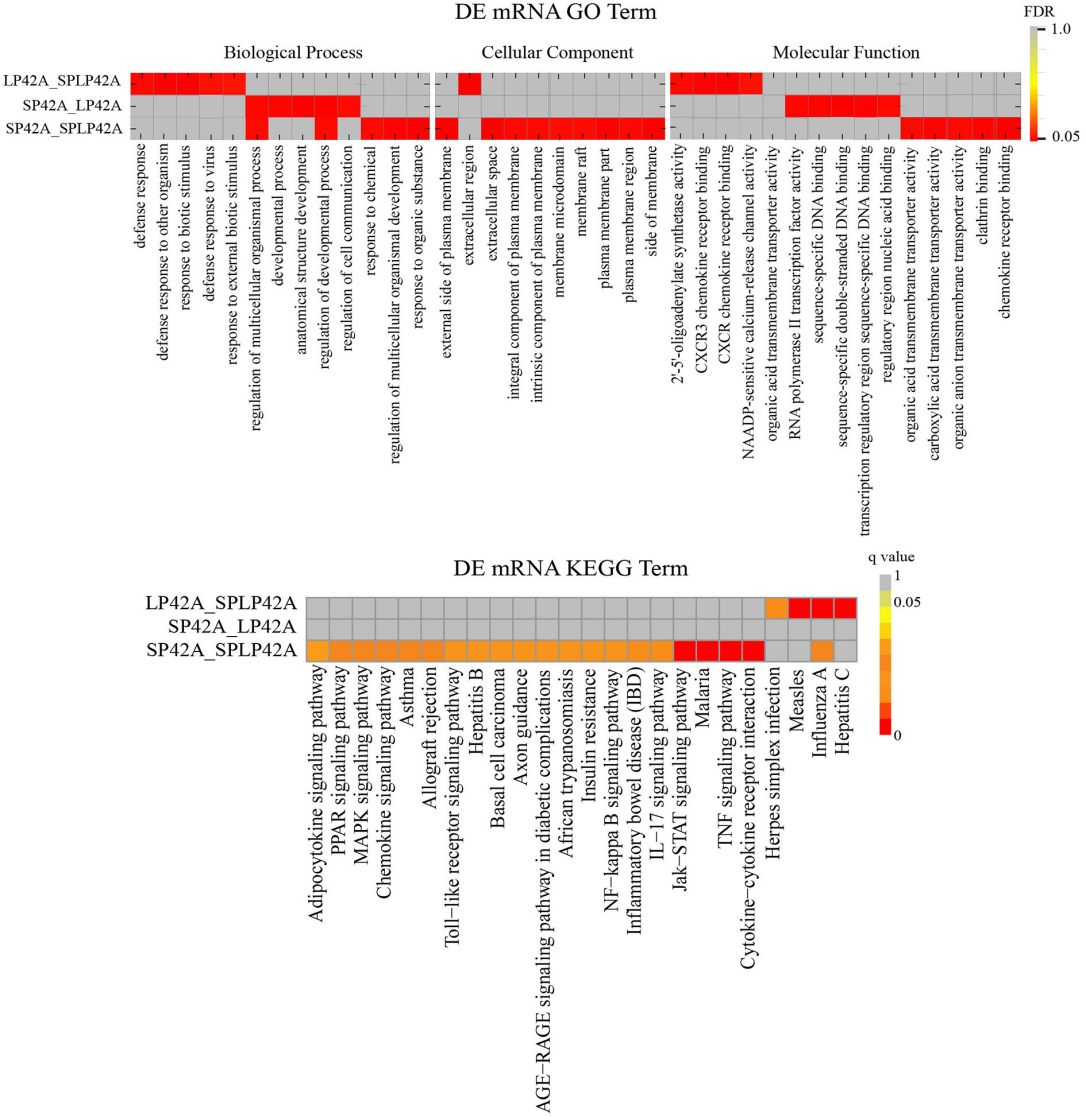
Functional enrichment analysis of DEGs. The upper part of the picture shows the enrichment results of GO and the lower part shows the enrichment results of KEGG.

To better understand the potential functions of the DEGs and target genes of DEMs, GO term and KEGG pathway analyses were performed. For DEGs, the three comparison groups we set up showed large differences. We found that the number of GO terms for biological processes significantly enriched in the three comparison groups was more than 50; in particular, the term number in the SP42 vs. SPLP42 comparison group was as high as 665. We selected only the top five terms in each group to draw the resulting chart. Second, we found a total of 10 terms in the three comparison groups regarding cellular components, so they were all drawn on the resulting graph. For the part of molecular function, except that we only found 4 significant terms in LP42 vs. SPLP42, the other two comparison groups chose the top five terms to draw the resulting map (Supplementary Table 9). Our main focus on BP terms included regulation of multicellular organismal process and regulation of the developmental process. The NAADP-sensitive calcium-release channel activity of the MF term also attracted our attention. The results of the KEGG pathway analysis are shown in Figure 5, and all the significantly enriched pathways found by the three comparison groups are plotted on the resulting map (Supplementary Table 10). The main pathways that attracted our attention were cytokine-cytokine receptor interactions, the TNF signaling pathway, the Jak-STAT signaling pathway and the MAPK signaling pathway.

Regarding the target genes of miRNAs, LP42 vs. SPLP42 (*n* = 119), SP42 vs. LP42 (*n* = 245) and SP42 vs. SPLP42 (*n* = 224) differential target genes were found (Supplementary Table 8). The functional enrichment analysis of miRNA target genes was divided mainly into known miRNAs and novel miRNAs. The results are shown in Table 3. In the taxonomic list of known miRNAs, we found only that there was significant enrichment of GO terms in the LP42 vs. SPLP42 comparison group, except that the first five terms were selected in the BP, and all the significantly enriched terms about CC and MF are shown in the rest of the list. We are most interested in Wnt-activated receptor activity, Wnt-protein binding and cytokine binding. However, the results of GO enrichment analysis of novel miRNA target genes showed that only 13 terms in the MF part of the SP42 vs. SPLP42 comparison group were significantly enriched (Supplementary Table 11). Finally, we were also surprised to find that there was no significant enrichment of pathways in either known miRNAs classification or novel miRNAs classification in the three comparison groups in the KEGG enrichment analysis. However, there were some pathways with *p* ≤ 0.05, not Q ≤ 0.05 (Supplementary Table 12).

**Table 3:** Functional enrichment analysis of target gene of DEMs.

**Target gene of know miRNA**				**Target gene of novel miRNA**			
**Term_name (LP42-SPLP42)**	**Accession**	**FDR**	**Type**	**Term_name (SP42-SPLP42)**	**Accession**	**FDR**	**Type**
DNA methylation on cytosine within a CG sequence	GO:0010424	0.021	BP	oligo-1,6-glucosidase activity	GO:0004574	0.001	MF
Cerebellum vasculature morphogenesis	GO:0061301	0.021		alpha-1,4-glucosidase activity	GO:0004558	0.003	
Cellular response to lipid	GO:0071396	0.021		alpha-glucosidase activity	GO:0090599	0.006	
Progesterone secretion	GO:0042701	0.021		hydrolase activity, hydrolyzing O-glycosyl compounds	GO:0004553	0.009	
Steroid hormone secretion	GO:0035929	0.021		hydrolase activity, acting on glycosyl bonds	GO:0016798	0.015	
Cell surface	GO:0009986	0.047	CC	glucosidase activity	GO:0015926	0.015	
DNA-methyltransferase activity	GO:0009008	0.008	MF	lysozyme activity	GO:0003796	0.022	
DNA (cytosine-5-)-methyltransferase activity, acting on CpG substrates	GO:0051718	0.008		peptidoglycan muralytic activity	GO:0061783	0.033	
DNA (cytosine-5-)-methyltransferase activity	GO:0003886	0.008		rRNA (uridine-N3-)-methyltransferase activity	GO:0070042	0.039	
Unmethylated CpG binding	GO:0045322	0.009		beta-fructofuranosidase activity	GO:0004564	0.039	
Wnt-activated receptor activity	GO:0042813	0.035		UDP-N-acetylglucosamine-dolichyl-phosphate N-acetylglucosaminephosphotransferase activity	GO:0003975	0.039	
Wnt-protein binding	GO:0017147	0.037		sucrose alpha-glucosidase activity	GO:0004575	0.039	
Cytokine binding	GO:0019955	0.044		phospho-N-acetylmuramoyl - pentapeptide-transferase activity	GO:0008963	0.039	

*The above GO term includes the significantly enriched term in the analysis results of all the comparison groups, except for the BP group in the known miRNA section, which ranks in the top 5. The absence of the comparison group name indicates that the comparison group has no significant enrichment of term*.

### Analysis of integrated miRNA–mRNA co-expression network

**Figure 6:**
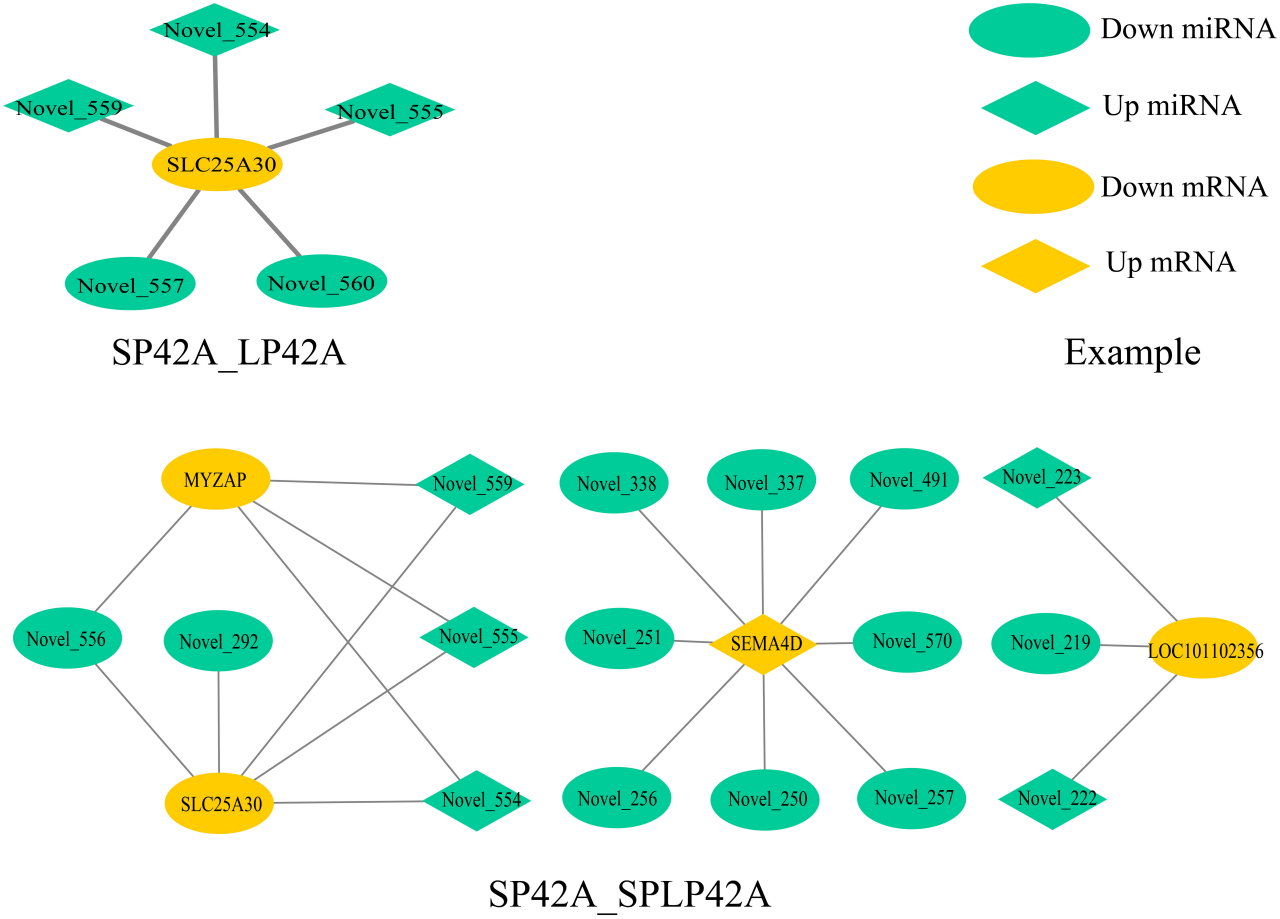
Overview of mRNA–miRNA networks. Different colors represent DEM (green) and DEG (yellow), and different shapes represent different modes of regulation.

To fully understand the potential roles of miRNAs, we built interactome networks using DEMs and their targets (DEGs). In total, 16 DE miRNAs (novel miRNAs) in SP42 vs. LP42 were predicted to target 67 genes. An mRNA–miRNA co-expression network was then constructed, where 1 DEG was targeted by 5 novel miRNAs. Regarding SP42 vs. SPLP42, 35 novel DE miRNAs were predicted to target 61 genes. The other mRNA–miRNA co-expression network was then constructed, where 4 DEGs were targeted by 16 novel miRNAs (Figure 6; Supplementary Tables 8, 13).

### Data validation

**Figure 7:**
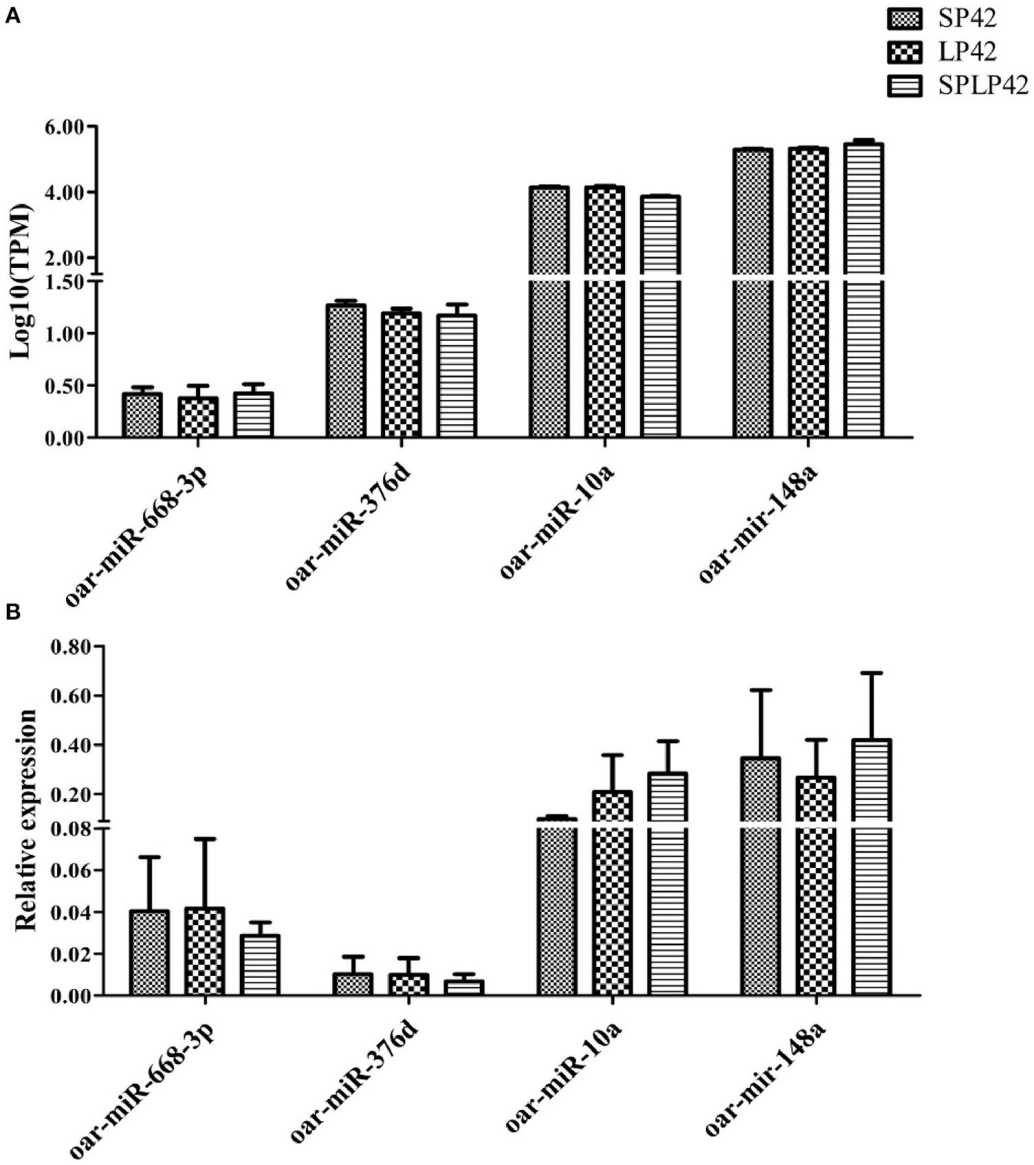
Quantitative verification results of miRNA. The relative expressions of 4 miRNA randomly selected in different individuals and among comparison groups were verified. Rectangles with different patterns represent RNA-seq **(A)** and qPCR **(B)** results, respectively.

To evaluate the accuracy of sequencing, qPCR was used to verify the RNA-seq data. The results showed that the expression pattern of miRNAs in sheep adrenal glands was similar to the expression pattern of sequencing (Figure 7), which proved the reliability of the data produced by RNA-seq.

## Discussion

The transcriptome is space-time specific, but it is a powerful means to mine key information in the process of animal genetic development. The purpose of this study was to establish an OVX model and assist the light-controlled environment to identify the key sheep miRNAs and differentially expressed genes affected by light changes. Our previous studies successfully obtained the miRNAome and mRNAome information of the hypothalamus and pituitary through a similar experiment (17, 22). Therefore, we selected an adrenal transcriptional group for detailed analysis based on the close relationship between HPG and the HPA axis and successfully identified new adrenal-specific miRNAs and differentially expressed genes.

In this study, a total of 861 miRNA and 17,712 coding protein genes were identified in the three comparison groups. Among the miRNAs we identified, novel miRNAs accounted for approximately 82.45% of the total number of miRNAs, and known miRNAs accounted for only 17.54%. Among the 151 mature known miRNAs, we obtained only 106 stem-loop sequences. Both the 5 p arm and 3 p arm of 47 stem-loop sequences produced mature miRNA. Both arms of a miRNA precursor may give rise to functional levels of mature miRNA (35, 36). The dominant products may change from species to species and have different tissue expression preferences, including normal vs. pathological tissue (37–43). At the same time, when identifying three biological repetitive samples in each treatment group, we found that except for SP42-1 and SP42-3, all the other individuals identified 106 stem-loop sequences. The stem-loop sequences that were not detected were oar-mir-654 (producing 3P mature miRNA) and oar-mir-1193 (producing 5p mature miRNA). mir-1193 has been found to inhibit the proliferation and invasion of cancer cells by directly acting on transmembrane 9 superfamily member 3 (*TM9SF3*) and insulin-like growth factor 2 mRNA binding protein 2 (*IGF2BP2*) (44, 45). In a mouse study, overexpression of mir-1193-5p was found to be able to increase the differentiation tendency of oligodendrocyte progenitor cells (46). Similarly, previous studies have found that mir-654 is significantly related to the development of a variety of malignant tumors, including lung cancer, rectal cancer, esophageal cancer and colon cancer (47, 48). Since we only detected the above two miRNAs in SP42-2 individuals, it is not possible to determine their relationship with the photoperiod and adrenal gland, so we will explore further.

After differential expression analysis, we initially identified 144, 454 and 506 DEGs and 48, 36 and 55 DE miRNAs (LP42 vs. SPLP42, SP42 vs. LP42 and SP42 vs. SPLP42). According to the grouping, the quantity distribution of DEGs was more consistent with the expectation of the experiment; that is, the number of differentially expressed genes in the SP42 vs. LP42 and SP42 vs. SPLP42 comparison groups was greater than the number of differentially expressed genes in the LP42 vs. SPLP42 comparison group. However, the results of miRNA analysis showed that there were the fewest DEMs in the SP42 vs. LP42 comparison group. When studying the data of the sheep pituitary transcriptome after different photoperiod treatments, with the increase in long photoperiod maintenance days, the number of differentially expressed genes and differentially expressed lncRNAs were found to increase significantly compared with the short photoperiod (22). What causes the number of differential miRNAs to appear in the small probability of the significant difference treatment group needs further analysis. Similarly, during a miRNA study of sheep hypothalamus, significant expression of the let-7 and oar-miRNA-200 families was found (17), and some studies also proved that those identified miRNAs were differentially expressed in seasonal and non-seasonal sheep breeds (49). However, it is unfortunate that although the let-7 and oar-miRNA-200 families were detected in this study, there was no differential expression in different comparison groups. In our known miRNA identification of sheep adrenal glands and Small Tail Han sheep hypothalamus (17), only six miRNAs (oar-miR-544-3p, oar-miR-411b-5p, oar-miR-376e-3p, oar-miR-376d, oar-miR-376b-3p, oar-miR-376a-3p) were specifically expressed in the adrenal gland, and one (oar-miR-1193-3p) was specifically expressed in the hypothalamus. The rest can be detected in both tissues. In addition, several miRNAs, such as miRNA-200 family members, were thought to be conserved in the hypothalamus of mice (16, 50), rats (51), zebrafish (52), and sheep (17). However, to sum up, it may be conserved in the species, not in sheep breeds and the tissue; perhaps it is just the wrong time to monitor.

All the DEGs were subjected to GO functional enrichment and KEGG enrichment analysis according to the set comparison group. Because there were too many enrichment items in the BP part and the MF part, we chose the top five terms that were significantly enriched. In the BP section, we find that there are coincident entries GO:0051239 and GO:0050793 in the SP42 vs. LP42 and SP42 vs. SPLP42 comparison groups. The overlapping genes *GRHL2* (grainy head-like protein 2 homologs), *CENPF* (centromere protein F) and *FGF16* (fibroblast growth factor 16) were found by further analysis of the above two terms. *GRHL2* is one of three mouse homologs of *Drosophila Grainyhead* (53) and is expressed in diverse embryonic epithelial tissues during development (53, 54). *GRHL2* and its paralogue *Grhl3* play essential roles in neural tube closure in mice (55–58). Data indicate that a conserved *GRHL2*-coordinated gene network controls trophoblast branching morphogenesis, thereby facilitating the development of the site of feto-maternal exchange (59). Normal placental development is a key factor in reproductive success, so whether screening this gene in our different photoperiod comparison groups indicates that placental development is affected by photoperiod is unknown. Therefore, it is very important to explore its function in the process of seasonal reproduction of sheep. Another key gene, *CENPF*, related to early embryonic development was also screened out. *CENPF* is a member of the centromere protein family that regulates chromosome segregation during mitosis (60, 61). Zhou et al. used mice to study and indicated that farnesylation plays a key role during *CENPF* degradation and localization in early embryos. At the same time, a knockout test of the *CENPF* gene showed that the gene is very important for early embryos (62). The fibroblast growth factor 16 (*FGF16*) gene is correlated with oocyte quality (63). In summer, when oocyte quality is low, the expression of *FGF16* is low. Conversely, in winter, when oocyte quality is high, the expression of *FGF16* is high. We found that the expression of the *FGF16* gene was upregulated in the comparative group of SP42 vs. LP42, indicating that in adrenal tissue, the expression of the *FGF16* gene under a long photoperiod was nearly 10 times lower than that under short photoperiod.

Our experimental analysis results are not ideal, whether it is the distribution of differential miRNAs in different comparison groups or the functional enrichment analysis of GO and KEGG of DEGs or the target genes of DEMs. However, the key comparison group SP42 vs. LP42 we set up has only made exciting discoveries in the process of GO enrichment analysis of mRNA. All the significantly enriched KEGG items of DEGs or target genes of DEMs are shown in the results section, but the SP42 vs. LP42 comparison group unexpectedly did not have any significant entries. Therefore, we need to further construct a miRNA–mRNA coexpression network to study interaction regulation and mine gene expression regulation patterns. We identified the overlapping core gene *SLC25A30* in the SP42 vs. LP42 and SP42 vs. SPLP42 comparison groups. SLC25A30 is a member of the mitochondrial transporter family (64). Mitochondrial transporters for inorganic anions/malate (SLC25A30), thiamine pyrophosphate (TPP) (SLC25A19) and iron (SLC25A28) are also considered conceptus-induced IFNT-dependent endometrial DEGs and are increased in endometrial epithelial cells by IFNT (65, 66). A study by Gorgoglione et al. (67) suggests that SLC25A30 may have the function of exporting sulfite and thiosulfate or transporting malate acid, which may contribute to the flow of malate–aspartate acid in mitochondria. Importantly, malic acid and malate dehydrogenase is present in the intrauterine environment of cattle, and malic acid has been shown to affect the early embryonic development of hamsters (68–70). *SLC25A30* gene expression has been reported to be upregulated by cellular oxidative stress, and our study may also be a sign of increased oxidative stress (71). Novel miRNA554, novel miRNA555 and novel miRNA559 are the common miRNAs that regulate the core gene in the two comparison groups (SP42 vs. LP42 and SP42 vs. SPLP42). These three miRNAs are located on chromosome 7 and belong to the same miRNA cluster according to location analysis. However, the specific functions need to be further analyzed.

These results suggest that several key DEGs and DEMs in the adrenal gland are directly or indirectly involved in the process of photoperiod-changing reproductive activity, and further study of gene/miRNA knockout or overexpression is helpful for us to understand their real function in female reproductive traits.

## Conclusions

We successfully obtained the mRNA and small RNA data of adrenal gland tissue samples of Sunite sheep under different photoperiod treatments by RNA sequencing and bioinformatics analysis. The key candidate genes of photoperiod affecting reproduction, such as *GRHL2, CENPF, FGF16* and *SLC25A30*, were confirmed. The miRNA (oar-miR-544-3p, oar-miR-411b-5p, oar-miR-376e-3p, oar-miR-376d, oar-miR-376b-3p, oar-miR-376a-3p) was specifically expressed in adrenal tissue. The predicted mRNA-miRNA pairs (*SLC25A30* regulated by Novel miRNA554, Novel miRNA555 and Novel miRNA559) showed significant differences in SP42 vs. the other two groups, indicating that the relationship may play an important role in the process of different photoperiod affecting adrenal function. Our results provide a new perspective for the study of sheep reproduction and help to deepen the understanding of ovine reproduction.

## Data availability statement

The datasets presented in this study can be found in online repositories. The names of the repository/repositories and accession number(s) can be found below: https://www.ncbi.nlm.nih.gov/sra/PRJNA756142; https://www.ncbi.nlm.nih.gov/sra/PRJNA811389.

## Ethics statement

The animal study was reviewed and approved by the Animal Experimental Welfare Ethics Committee of the Institute of Animal Sciences, Chinese Academy of Agricultural Sciences.

## Author contributions

QiuL and MC designed the research. XD wrote the paper and performed the study. RD, XD, and QinL collected the data. XD and XH analyzed data. MC revised the final manuscript. All authors reviewed the manuscript and approved the final version.

## Funding

This work was financially supported by National Natural Science Foundation of China (32172704), China Agriculture Research System of MOF and MARA (CARS-38), and the Agricultural Science and Technology Innovation Program of China (CAAS-ZDRW202106 and ASTIP-IAS13).

## Conflict of interest

The authors declare that the research was conducted in the absence of any commercial or financial relationships that could be construed as a potential conflict of interest.

## Publisher's note

All claims expressed in this article are solely those of the authors and do not necessarily represent those of their affiliated organizations, or those of the publisher, the editors and the reviewers. Any product that may be evaluated in this article, or claim that may be made by its manufacturer, is not guaranteed or endorsed by the publisher.

## Abbreviations

mRNA: message RNA
miRNA: microRNA
HPA: The hypothalamic–pituitary–adrenal
LP: Long photoperiod
SP: Short photoperiod
GO: Gene Ontology
KEGG: Kyoto Encyclopedia of Genes and Genomes
LP42: The LP lasts for 42 days
SP-LP42, The SP lasts for 42 days: and then convert it to LP for 42 days
SP42: The SP lasts for 42 days
*GRHL2*: grainy head-like protein 2 homologs
*CENPF*: centromere protein F
*FGF16*: Fibroblast growth factor 16
*SLC25A30*, solute carrier family 25: member 30.
