# Supplementary material for: Identification of photoperiod-induced specific miRNAs in the adrenal glands of Sunite sheep (Ovis aries)
Source: Front Vet Sci. 2022 Jul 22;9:888207. doi: 10.3389/fvets.2022.888207 (PMC9354845; doi:10.3389/fvets.2022.888207)
Supplement: Supplementary file 1 [file Data_Sheet_1.ZIP › Supplementary file/Supplementary Material.docx]

Supplementary Material

**Supplementary Table S1 |** Overview of the quality control of miRNA and mRNA reads generated from the adrenal gland.

**Supplementary Table S2 |** The fragments per kilobase per million mapped fragments (FPKM) values of mRNAs identified in nine sample。

**Supplementary Table S3 |** The region of identified mRNAs in SP42A (short photoperiod for 42 days; n = 3), LP42A (long photoperiod for 42 days; n = 3) and SPLP42A (short photoperiod for 42 days followed by a long photoperiod for 42 days; n = 3) from the genome perspective.

**Supplementary Table S4 |** Chromosome distribution of identified mRNA and novel miRNA in SP42A (short photoperiod for 42 days; n = 3), LP42A (long photoperiod for 42 days; n = 3) and SPLP42A (short photoperiod for 42 days followed by a long photoperiod for 42 days; n = 3) from the adrenal gland.

**Supplementary Table S5 |** The transcripts per million (TPMs) value of miRNAs identified in nine sample and the hypothalamus of Small Tail Han sheep.

**Supplementary Table S6 |** The identification involving diverse RNAs in nine sample

**Supplementary Table S7 |** Identification of differentially expressed mRNA and miRNA in different comparison groups

**Supplementary Table S8 |** The prediction of target genes of miRNAs in in different comparison groups

**Supplementary Table S9 |** GO enrichment annotation for mRNAs in terms of their molecular function (MF), biological process (BP), and cellular component (CC) level in different comparison groups

**Supplementary Table S10 |** KEGG enrichment annotation for mRNAs in different comparison groups

**Supplementary Table S11 |** GO enrichment annotation for target genes of differentially expressed miRNAs in different comparison groups.

**Supplementary Table S12 |** KEGG enrichment annotation for target genes of differentially expressed miRNAs in different comparison groups.

**Supplementary Table S13 |**

**Supplementary Table S14 |** Real-time quantitative polymerase chain reaction primers of the amplification products of the selected miRNAs and housekeeping genes.
